# Supplementary material for: The effect of calcaneus and metatarsal head offloading insoles on healthy subjects’ gait kinematics, kinetics, asymmetry, and the implications for plantar pressure management: A pilot study
Source: PLoS One. 2024 May 17;19(5):e0303826. doi: 10.1371/journal.pone.0303826 (PMC11101073; doi:10.1371/journal.pone.0303826)
Supplement: S1 File — This file can be accessed in Mendeley data (DOI: 10.17632/hfz543gjc7.1). Two folders are included in the ‘S1_File’ folder. (DOCX) [file pone.0303826.s001.docx]

Unprocessed data of plantar pressure, ground reaction force and gait kinematics can be found using the link below:

[The effect of calcaneus and metatarsal head offloading insoles on healthy subjects’ gait kinematics, kinetics, asymmetry, and the implications for plantar pressure management. - Mendeley Data](https://data.mendeley.com/datasets/hfz543gjc7/1)





Fig 1. Hierarchy of the folder of unprocessed data of plantar pressure, ground reaction force and gait kinematics. 'Qualisys' folder encompasses 237 files containing jointed ground reaction force and gait kinematics data. Meanwhile, the 'Tekscan' folder includes 180 files containing plantar pressure data. All files are denoted by a nomenclature adhering to the convention: ‘subject number’ _ ’offloading condition’ _ ’walking speed/static’, where the ‘static’ means the static model data. SCO means small calcaneus offloading insole, LCO means larger calcaneus offloading insole, SMHO means small metatarsal head offloading insole, LMHO means large metatarsal head offloading insole, and LCOBS means both sides large calcaneus offloading insole.
