# Supplementary material for: The effect of calcaneus and metatarsal head offloading insoles on healthy subjects’ gait kinematics, kinetics, asymmetry, and the implications for plantar pressure management: A pilot study
Source: PLoS One. 2024 May 17;19(5):e0303826. doi: 10.1371/journal.pone.0303826 (PMC11101073; doi:10.1371/journal.pone.0303826)
Supplement: S1 Table — (DOCX) [file pone.0303826.s002.docx]

| Subject | Slow walking speed (m/s) | Normal walking speed (m/s) | Fast walking speed (m/s) |
| --- | --- | --- | --- |
| HF1 | 0.8 | 1.0 | 1.2 |
| HF2 | 1.0 | 1.2 | 1.4 |
| HF3 | 0.6 | 0.8 | 1.0 |
| HF4 | 0.9 | 1.1 | 1.3 |
| HF5 | 0.8 | 1.0 | 1.2 |
| HF6 | 0.8 | 1.0 | 1.2 |
| HF7 | 0.75 | 0.95 | 1.15 |
| HF8 | 1.0 | 1.2 | 1.4 |
| HF9 | 0.9 | 1.1 | 1.3 |
| HF10 | 1.0 | 1.2 | 1.4 |
