# Supplementary material for: The effect of calcaneus and metatarsal head offloading insoles on healthy subjects’ gait kinematics, kinetics, asymmetry, and the implications for plantar pressure management: A pilot study
Source: PLoS One. 2024 May 17;19(5):e0303826. doi: 10.1371/journal.pone.0303826 (PMC11101073; doi:10.1371/journal.pone.0303826)
Supplement: Attachment — Submitted filename: Response to Reviewers.docx [file pone.0303826.s003.docx]

School of Engineering

The University of Manchester

Engineering Building A

Manchester M13 9PL

+44(0)161 529 3401

[Jiawei.shuang@manchester.ac.uk](mailto:Jiawei.shuang@manchester.ac.uk)

[www.mace.manchester.ac.uk](http://www.mace.manchester.ac.uk)

21 Feb 2024

Dear Dr Jianhong Zhou

**Re: PLOS ONE Decision: Revision required [PONE-D-23-30055] - [EMID:db690a3d5d331f19]**

Thank you for giving us the opportunity to submit a revised draft of our manuscript titled ‘**The effect of calcaneus and metatarsal head offloading insoles on healthy subjects’ gait kinematics, kinetics, asymmetry, and the implications for plantar pressure management**’ to PLOS ONE.

We appreciate the time and effort that you and the reviewers have dedicated to providing your valuable feedback and comments on our manuscript. We have tried to incorporate changes to reflect the suggestions provided by the reviewers.

Here is a point-by-point response to the reviewer’s comments and concerns. Lines quoted are for the ‘Revised Manuscript with Track Changes’.

**Comments from the Review #1:**

**Summary of Comments:**

This work tested 6 types of insoles, including a control insole and off-loading insoles, on the treadmill walking. This study analyzed plantar pressure and walking kinematics. Even this study performed in healthy subjects but it aimed to used the results for diabetic patients. There are several concerns needed to be clarified.

**Response:**

Thank you for taking the time to read our manuscript. We really appreciate the feedback and

insightful comments on our manuscript. We have tried to address your concerns on comments for diabetic patients in the responses to the comments below.

**Comment 1:**

Did the authors measure the arch index of the participants? Or any contact area is analyzed with the F-scan.

**Response:**

Thank you for highlighting this. We have now calculated the average arch index and total foot contact area of the six insole conditions for ten subjects at three walking speeds. We have now given the method detail in line 181 (page 8), showed the result in line 292 (page 14), included the table in line 298 (page 14) and in line 450 (page 20) of our manuscript, discussed the sample size limitation in line 353 (page 17).

See the highlighted sentences below for changes and the table:

Added to method section (line 181):

“*The arch index was calculated from normal plantar pressure measurements taken with the F-scan system, using the method outlined in reference [24]. In addition, the total contact area of the foot was recorded and exported.”*

Added to the Results section (line 292):

“*Table 4 shows the mean arch index and total contact area. P-values were calculated for the five offloading conditions and the control condition for the 10 subjects at three walking speeds (S6 Table). There was a statistical trend that showed decreases in the arch index for the SCO condition during fast walking (-7%, P<0.05), the SMHO condition during fast walking (-9%, P<0.05), and the LMHO condition during slow and normal walking (-9%, P<0.05; -14%, P<0.05). A statistical trend showed decreases in the total contact area for the SCO condition during slow walking (-4%, P<0.05) and the LMHO condition during slow and normal walking (-5%, P<0.05; -6%, P<0.05).*

Added Table 4 to the main text (line 298):

*Table 4. The mean arch index and total contact area of five offloading conditions and the control condition for 10 subjects at three walking speeds.*

| Condition | Walking speed | Arch index (*mean[std]*) | Contact area (mm^2, *mean[std]*) |
| --- | --- | --- | --- |
| Control  (Control flat insole) | Slow | 0.22[0.04] | 9387[1612] |
|  | Normal | 0.21[0.06] | 9347[1756] |
|  | Fast | 0.21[0.05] | 9489[1724] |
| SCO  (Small calcaneus offloading insole) | Slow | 0.21[0.06] | 9004[1531] |
|  | Normal | 0.21[0.06] | 9259[1781] |
|  | Fast | 0.20[0.06] | 9212[1750] |
| LCO  (Larger calcaneus offloading insole) | Slow | 0.22[0.05] | 9607[1607] |
|  | Normal | 0.21[0.05] | 9545[1511] |
|  | Fast | 0.21[0.05] | 9752[1745] |
| SMHO  (Small metatarsal head offloading insole) | Slow | 0.20[0.05] | 9219[1513] |
|  | Normal | 0.20[0.06] | 9407[1745] |
|  | Fast | 0.20[0.05] | 9429[1557] |
| LMHO  (Large metatarsal head offloading insole) | Slow | 0.21[0.04] | 9379[1451] |
|  | Normal | 0.19[0.03] | 9258[1129] |
|  | Fast | 0.19[0.05] | 9554[1401] |
| LCOBS  (Both sides large calcaneus offloading insole) | Slow | 0.22[0.05] | 9412[1273] |
|  | Normal | 0.21[0.04] | 9444[1312] |
|  | Fast | 0.20[0.04] | 9573[1312] |

“

Added to the discussion section (line 353):

“*Our sample size was small and chosen for convenience sampling, as is common for pilot studies of this type [20] therefore our results may not be statistically significant. However, the results of this study do show some indicative relationships between gait kinematics, and plantar pressure for offloading insole conditions but further work would be needed to determine if these relationships are significant.*”

Added to the supporting information section (line 450):

“*S6 Table. The mean arch index and total contact area along with the percentage difference and P-value between five offloading conditions and the control condition for 10 subjects at three walking speeds.*

| Percentage difference with the P-value | | | |
| --- | --- | --- | --- |
| Condition | Walking speed | Arch index (Control value, no unit;  *difference from control, % and [P-value]*) | Contact area  (Control value, mm^2;  *difference from control, % and [P-value]*) |
| Control  (Control flat insole) | Slow | 0.22 | 9387 |
|  | Normal | 0.21 | 9347 |
|  | Fast | 0.21 | 9489 |
| SCO  (Small calcaneus offloading insole) | Slow | -2%[0.90] | -4%[<0.05] |
|  | Normal | -2%[0.87] | -1%[0.56] |
|  | Fast | -7%[<0.05] | -3%[0.10] |
| LCO  (Larger calcaneus offloading insole) | Slow | 3%[0.28] | 1%[0.53] |
|  | Normal | 2%[1.00] | 1%[0.94] |
|  | Fast | 1%[0.81] | 1%[0.38] |
| SMHO  (Small metatarsal head offloading insole) | Slow | -7%[0.15] | -3%[0.17] |
|  | Normal | -4%[0.26] | -1%[0.53] |
|  | Fast | -9%[<0.05] | -2%[0.22] |
| LMHO  (Large metatarsal head offloading insole) | Slow | -9%[<0.05] | -5%[<0.05] |
|  | Normal | -14%[<0.05] | -6%[<0.05] |
|  | Fast | -14%[0.08] | -3%[0.14] |
| LCOBS  (Both sides large calcaneus offloading insole) | Slow | 0%[0.28] | -1%[0.53] |
|  | Normal | 1%[1.00] | 0%[0.73] |
|  | Fast | -3%[0.30] | 0%[0.69] |

**Comment 2:**

As this study used 3 measurement tools (F-scan, motion capture and force plate), how did the authors sync the signals from three measurement?

**Response:**

Thank you for highlighting this. We achieved synchronization between the two force plates and the motion capture system through a hardwired connection. We used the initial heel strike as an event to synchronize the in-shoe pressure measurement system with both the force plates and the motion capture system. We added these details to line 148 (page 6) of our manuscript.

See the shaded sentences below for changes and the figure:

*“…and F-Scan research (v7.00 -19), respectively. Synchronization between the two force plates and the motion capture system was achieved through a hardwired connection. The initial heel strike was utilised as a common event to synchronize the in-shoe pressure measurement system with both the force plates and the motion capture system.”*

**Comment 3:**

It is better to provide the pattern of gait cycles on the treadmill to demonstrate PTI.
**Response:**

Thank you for your suggestions. We added the figure to provide this information in line 249 (page 11) and line 263 (page 12) of our manuscript.

See the shaded sentences below for changes and the figure:

***“****Fig 6 presents the plantar pressure and* *the pressure time integral in the regions of interest at the calcaneus and metatarsal heads during one gait cycle of ten subjects walking at normal speed.* *Fig 6 shows that the SCO, LCO, and LCOBS insoles decreased the peak plantar pressure in calcaneus region, while no difference of metatarsal heads peak plantar pressure was observed when utilising the SMHO and LMHO insoles.* *Additionally, all the conditions show differences in peak plantar pressure profiles across the gait cycle for either the calcaneus or metatarsal head region.*

*. Table 2 shows the mean…”*

*“Fig 6: The plantar pressure and the pressure time integral in the regions of interest at the calcaneus and metatarsal heads during one gait cycle of ten subjects walking at normal speed. The black circles and squares indicate the locations of peak plantar pressure at the calcaneus and metatarsal heads regions of interest respectively, while the blue and red shadows represent the pressure time integral at the calcaneus and metatarsal heads regions of interest, respectively.”*

**Comment 4:**

Please provide the plantar pressure distribution from F-scan of each insole. These information will give clear picture of edge effect.
**Response:**

Thank you for your suggestions. We add the figure of the plantar pressure distribution from F-scan in line 225 (page 10) and line 252 (page 10) of our manuscript.

See the shaded sentences below for changes and the figure:

*“Fig 5 presents the average peak plantar pressure (APPP) of one subject under five offloading conditions and a control condition, each recorded at a normal walking speed. Table 1 shows…”*

*“Fig 5. The average peak plantar pressure of one subject under five offloading conditions and a control condition, each recorded at a normal walking speed. The red dash cycle is the location of offloading aperture.”*

**Comment 5:**

Why did the authors choose 10 cycles to represent in this study? How long did the participant walk on the treadmill?
**Response:**

Thank you for pointing this out. Participants walked for 10 minutes in total on the treadmill for three speeds of each condition. For each walking speed, 20 seconds was used to adjust the treadmill speed and the remaining three minutes were used to walk at constant speed. The ten gait cycles were taken from the middle portion of the three-minute constant speed walking. From observation of the data variability within this period was extremely low. Other researchers have typically used ten gait cycles for their analysis, see ref Shi et al. (2022) and Moe-Nilssen, R (1998). Furthermore, we added a better explanation on this within the text in line 159 (page 7) of our manuscript.

See the shaded sentences below for changes:

*“normal walking speed (NWS) which was their self-selected walking speed to reflect their preferred walking pace. Subjects then walked on the treadmill at a slow speed (NWS - 0.2 m/s), then normal speed (NWS) and finally fast speed (NWS + 0.2 m/s) for 3 minutes and 20 seconds each (total duration 10 minutes). The participants started from rest on the treadmill and the speed was changed through normal, fast and slow walking speeds.”*

**References:**

Shi, Q.Q., Li, P.L., Yick, K.L., Li, N.W. and Jiao, J., 2022. Effects of contoured insoles with different materials on plantar pressure offloading in diabetic elderly during gait. Scientific Reports, 12(1), p.15395.

Moe-Nilssen, R., 1998. Test-retest reliability of trunk accelerometry during standing and walking. Archives of physical medicine and rehabilitation, 79(11), pp.1377-1385.

**Comment 6:**

There is a typo error on line 263 (page 12) for 10 subjects. It typed as 1o which is "o" character, not "0" number.
**Response:**

Thank you for pointing this out. We correct this typo in line 267 (page 12) of our manuscript.

See the shaded sentences below for changes:

*“Table 2. The mean pressure time integrals and shank distal end velocity of five offloading conditions and the control condition at the region of foot (RoF) and region of interest (RoI) for 10 subjects at three walking speeds.”*

**Comment 7:**

As the authors addressed about the limitations of this study, how can the finding be translated for the diabetic foot ulceration management? It is mandatory to do a further study in the diabetic patients as the authors mentioned but what the finding in healthy subjects can give to the aspect of the insole design.

**Response:**

Thank you we agree that there is a need for further study in diabetic subjects. This study was initially conducted on healthy participants before diabetic participants, to allow for the refinement of insole designs without risking harm to diabetic participants. The behaviours observed in healthy participants can offer indications of how diabetic participants might respond and provide control data for comparisons and better understanding of gait metrics either causing or resulting from diabetes. This is the first study to jointly measure gait kinematics and plantar pressure changes caused by offloading insoles, which remove insole material from high-risk regions. Some offloading insole configurations may increase heel strike and toe-off velocity, potentially leading to increases in plantar pressure. Although the current findings do not directly apply to diabetic foot ulcer management or diabetic subjects, they are indicative of general gait changes which will help in guiding the development and evaluation of insoles that could significantly reduce the risk of diabetic foot ulceration.

Furthermore, we added a better explanation on this within the text in line 357 (page 17) of our manuscript.

See the shaded sentences below for changes:

*“results of Penny et al. [10] also observed the inter-subject variability of peak plantar pressure.* *This study involved healthy participants to assess insole design safety and effect on gait kinematics and plantar pressure. This information could then be used in future studies on diabetic populations to ensure a safe and non-harmful approach. Although the current findings do not directly apply to diabetic foot ulcer management or diabetic subjects, they are indicative of general gait changes which will help in guiding the development and evaluation of insoles that could significantly reduce the risk of diabetic foot ulceration. This effect (increasing heel strike and toe off speed) may be increased in a diabetic population which is likely to lead to balance problems in gait.”*

**Comments from the Review #2:**

**Summary of Comments:**

Generally, the topic of this study look interesting and the outcome in this study is strong receiving from the laboratory. My major concern is the statistical analysis of this study, there is no clear information regarding this, which affect the result of this study. Any details in the discussion part should be described in the correlation with the result, the result part did not show the p-vale, anyways the authors use the word “significant” to explain the result. With regards to specific comments in each part, my comments are described following.

**Response:**

Thank you for this insightful comment. We agree and more detailed information on the statistical analysis has been added to the paper.

**Comment 1:**

INTRODUCTION: “Specially designed insoles can be part of an effective DFU prevention strategy which includes maintaining mobility and physical activity as part of a holistic management package [6,7].”
Reviewer: How about the other interventions? The authors should imply general interventions for DFU for a few sentences before raising the insole as the important intervention.
**Response:**

Thank you for pointing this out. We add a few sentences to introduce the general interventions for DFU in line 41 (page 2) of our manuscript.

See the shaded sentences below for changes:

*“General interventions for diabetic foot ulcer (DFU) include self-care practices, education and self-management, the employment of footwear and orthotic devices, along with diverse clinical treatment strategies [6]. Among these, the use of appropriate footwear stands out as a critical element in the prevention of DFU [6]. Specially designed insoles…”*

**Comment 2:**

INTRODUCTION: “Lin et al. [8] evaluated the average peak plantar pressure in the forefoot region of an insole with removable plugs and a support arch using a pedar in-shoe plantar pressure measurement system on diabetic patients”
Reviewer: Please explain more about the effects of DFU on increasing forefoot plantar pressure before describing the results of Lin et al. study.
**Response:**

Thank you for your suggestion. We add a sentence to discuss the relationship between the increasing forefoot pressure and the DFU in line 45 (page 2) of our manuscript.

See the shaded sentences below for changes:

*”* *Significantly higher peak forefoot pressures are observed in diabetic subjects, for example researchers report 608 kPa and 373 kPa in diabetics with severe and moderate neuropathic, compared to294 kPa and 323 kPa in the mild neuropathic and nonneuropathic diabetic groups (P < 0.0001), suggesting an important role in the etiology of the diabetic foot condition [11]. Lin et al. [9] evaluated…”*

**Comment 3:**

INTRODUCTION: “Previous research has clearly shown that offloading insoles are effective at reducing normal plantar pressure in a specific region, with some impact on surrounding areas”
“To the authors’ knowledge, the effect of offloading insoles on gait kinematics, kinetics and plantar pressure has not been investigated.
Reviewer: The authors have already explained about the edge effects of offloading insole. Please describe more in the research gap about how to solve this problem in the present study.
**Response:**

Thank you for pointing this out. Our study was not designed to be an intervention to solve the problem of DFU. Rather it aimed to investigate the relationship between offloading regions in the insole and their effect on both the plantar pressure and the gait kinematics. But we have commented on solutions of the edge effect problem from the literature (Shualian et al. 2022 Chen et al 2003). We clarify this in line 78 (page 4) of our manuscript.

See the shaded sentences below for changes:

*“**This study aims to investigate the effect of offloading insoles on plantar pressure and their influence on the gait kinematics. To the authors knowledge this is the first study which jointly measures both plantar pressure and gait kinematics using offloading insoles. The results should help to guide the future development and evaluation of offloading insoles that could reduce the risk of high plantar pressure and altered gait styles in a diabetic population. This is a pilot study and therefore only aims to measure the effects of the insole intervention rather than to solve any issues observed. The objectives of this study are: (a) evaluating the offloading insoles effect on high risk region; (b) estimating the offloading effect on gait kinematics and kinetics; (c) examining the subjects gait asymmetry change; (d) and analysing the relationship between the gait kinematics, kinetics and plantar pressure.”*

**Comment 4:**

METHODS: “Ten participants were recruited for this experiment,”
Reviewer: Why the present study conduct only 10 healthy participants? How about the sample size calculation?
**Response:**

Thank you for pointing this out. As a proof-of-concept study, n > 6 allows for initial data to inform the need for a future feasibility study and to allow for refinement of the procedure prior to future data collection, incorporating data from usability and acceptability from the participants and research team. Ten participants were recruited for this experiment (sample size chosen for convenience sampling, pilot study similar to Bartolo et al. 2021). In the next study we will recruit at least 30 participants to achieve central limit theorem and to calculate point estimates and 95% confidence intervals. The feasibility phase will follow the recommendations from the CONSORT extension to randomised pilot and feasibility trials (PAFS) [Eldridge et al. 2016 & Eldridge et al. 2016]. PAFS emphasizes testing all aspects of data collection and processes of the intervention and measurement but warns against between-group testing of efficacy due to lack of statistical power. We add the detailed information to line 93 (page 4) of our manuscript.

See the shaded sentences below for changes:

*“Ten healthy participants (five male and five female) who did not have diabetes were recruited between 10th Sep 2022 and 1st October 2022 (sample size was chosen for convenience sampling similar to reference [20]).”*

**References:**

Bartolo, E., Giacomozzi, C., Coppini, D.V. and Gatt, A., 2021. The effect of induced joint restriction on plantar pressure during gait–a pilot study. Gait & Posture, 90, pp.23-28.

Eldridge, S.M., Lancaster, G.A., Campbell, M.J., Thabane, L., Hopewell, S., Coleman, C.L. and Bond, C.M., 2016. Defining feasibility and pilot studies in preparation for randomised controlled trials: development of a conceptual framework. PloS one, 11(3), p.e0150205.

Eldridge, S.M., Chan, C.L., Campbell, M.J., Bond, C.M., Hopewell, S., Thabane, L. and Lancaster, G.A., 2016. CONSORT 2010 statement: extension to randomised pilot and feasibility trials. bmj, 355.

**Comment 5:**

METHODS: “Ten healthy participants (five male and five female) who did not have diabetes were recruited”
Reviewer: The author should clarify the exclusion criteria. How about the history of lower-extremity fracture, other systemic conditions, the musculoskeletal symptoms of lower back and lower extremity etc.?
**Response:**

Thank you for your suggestion. We add the exclusion criteria in line 94 (page 4) of our manuscript.

See the shaded sentences below for changes:

*“The exclusion criteria for the study are as follows: participants under the age of 18; individuals with any type of movement disorder; those who experience pain while walking; individuals with broken skin on their foot; and anyone suffering from severe skin conditions, including eczema, skin allergies or any kind of foot problems. All participants…”*

**Comment 6:**

METHODS: “Six types of offloading insoles were manufactured including a control flat insole (Control), a small calcaneus offloading insole (SCO), a large calcaneus offloading insole (LCO), a small metatarsal head offloading insole (SMHO), a large metatarsal head offloading insole (LMHO), and a large calcaneus offloading insole for both feet (LCOBS) (Fig 1C). The LCOBS was the symmetric condition to compare to unilateral insoles (LCO).”
Reviewer: Please explain why the authors use the symmetric condition for only the calcaneus offloading, not the metatarsal head offloading.
**Response:**

Thank you for pointing this out. The aim of this study was to investigate the effect of offloading insoles on pressure offloading and their influence on the gait and balance. Offloading insoles are usually prescribed asymmetrically, therefore, our study focused on these asymmetric conditions. However, as we are interested on the effect on gait kinematics, we tested one symmetrical condition to understand if symmetry/asymmetry would have an effect. To ensure that testing time for the participants was not too long we had to limit the symmetrical condition to just one insole type and LCOBS was selected. We added the reason to the line 114 (page 5) of our manuscript.

See the shaded sentences below for changes:

*“**LCOBS was the symmetric condition with the LCO insoles in both left and right shoes. Although offloading insoles are normally prescribed asymmetrically, this study aims to understand the effect of offloading insoles on gait kinematics therefore this single symmetric condition was selected to study the effect of symmetrical insole conditions.”*

**Comment 7:**

METHODS: “The 1st and 2nd metatarsal heads location was defined from the mean anatomical locations measured from a small sample of 6 healthy participants which gave L1, W1, L2, and W2 are 71%, 85%, 74% and 63% respectively,”
Reviewer: Why the authors use the mean from 6 healthy participants, not from 10 participants.
**Response:**

Thank you for pointing this out. Insoles took 1-2 days to manufacture to allow for glue curing. So, to ensure that the testing was restricted to one visit to the gait lab for subjects, a pre-study to determine average dimensions for offloading location was conducted on six healthy volunteers. The six volunteers were not part of the ten subjects during the formal experiment. We add this detail in line 124 (page 6) of our manuscript.

See the shaded sentences below for changes:

*“****Offloading aperture location***

*Fig 1D shows the location of the calcaneus, 1st and 2nd metatarsal heads. The calcaneus location was 15% from the rear of the total foot length and central in the medial-lateral direction [24]. The 1st and 2nd metatarsal heads’ location was determined using a pre-study on six healthy volunteers (four male and two female). The mean and standard deviation of age, height and weight of the participants was 29.7 (7.6) years old, 179.8 (10.4) cm and 71 (20.8) kg respectively.* *The 1st and 2nd metatarsal heads’ location was defined by manually palpating their feet to locate the bony anatomy, which was then marked with a pen. The subject then stood on a sheet of paper to transfer the ink marks to the paper to enable accurate measurements. The mean values of L1, W1, L2, and W2 were 71%, 85%, 74% and 63% respectively, where L is the percentage of foot length and W is the percentage of the foot width (Fig 1D). Both the small and large calcaneus offloading aperture centres were located at the calcaneus location. The small metatarsal head offloading aperture centres were located at 1st metatarsal head location, and the large metatarsal head offloading aperture centres were located at the mid-point between 1st and 2nd metatarsal heads, which is 72.5% of the foot length and 74% of the foot width.”*

**Comment 8:**

METHODS: “A total of 16 markers were affixed to the diabetic shoes, excluding the body markers (Fig 2).”
Reviewer: The author should explain more in the discussion part about the advantage and disadvantage of the shoe-mounted marker and the skin-mounted marker.
**Response:**

Thank you for your suggestion. We discuss the shoe-mounted markers and skin-mounted markers in line 344 (page 16) of our manuscript.

See the shaded sentences below for changes:

*“The limitations of this study are the shoe-mounted markers, the generic insole, small sample size and healthy subjects. In this study, the limitation is recognised of using shoe-mounted markers, as they cannot represent the foot's movement inside the shoe, unlike skin-mounted markers which measure actual foot motion [28].* *However, we chose shoe-mounted markers to maintain the integrity of the footwear, considering its potential effect on gait. The measurement of foot motion will therefore have some inaccuracy, but the analysis of this study does not focus on foot motion, instead looks at ankle angle, therefore, the impact of using shoe-mounted markers is minimised. This study used…”*

**Comment 9:**

METHODS: “The data from the 10 participants were evaluated on an individual basis using the data from the individuals control insole condition as a baseline to compare kinematic and pressure differences.”
Reviewer: The author should clarify the statistical method that was used in this study. There is no clearly information about the method to compare the data among 6 conditions using the p-value.
**Response:**

Thank you for pointing out that. We have added the statistical method section in line 218 (page 9) of our manuscript.

See the shaded sentences below for changes:

*“****Statistical method***

*Statistical analyses were conducted using IBM SPSS Statistics (version 29.0.2.0, IBM SPSS, Inc., Chicago, IL, USA). Prior to analysis, A Shapiro-Wilk test was conducted to assess the normality of the data. The results of the Shapiro-Wilk test confirmed that the data were normally distributed. A paired t-test was then conducted to compare the plantar pressure and kinematic data between five offloading and one control insole conditions.*

**Comment 10:**
RESULTS: “Table 1 shows the mean average peak plantar pressure (APPP) and shank distal end velocity percentage difference between five offloading conditions and control condition at region of foot (RoF) and region of interest (RoI) (defined in Fig 3) of 10 subjects at three walking speeds.”
Reviewer: It would be better to describe the mean and sd of each outcome and re analyze the statistical difference with the p-value among the 6 conditions of insole.
**Response:**

Thank you for your suggestions. We rebuild the table according to your advice in line 226 (page 10), line 242 (page 10) and line 433 (page 19) of our manuscript. Additionally, we also added a figure to show the peak pressure profiles over the gait cycle (Fig 6).

See the shaded sentences below for changes:

*“**Table 1 shows the mean average peak plantar pressure at region of foot (RoF) and region of interest (RoI) (defined in Fig 3) and shank distal end velocity of five offloading conditions and the control condition for 10 subjects at three walking speeds. Percentage differences and P-values were calculated for the five offloading conditions and the control condition for 10 subjects at three walking speeds (S3 Table).* *Only LCO and LCOBS showed a decrease in APPP in the region of interest at calcaneus when walking at all three speeds (P<0.05). “*

*“Table 1. The mean average peak plantar pressure and shank distal end velocity of five offloading conditions and the control condition at the region of foot (RoF) and region of interest (RoI) for 10 subjects at three walking speeds.*

| Walking speed | Offloading condition | Velocity (m/s, *mean[std]*) | | APPP at RoF (kPa, *mean[std])* | | | APPP at RoI (kPa, *mean[std]*) | | |
| --- | --- | --- | --- | --- | --- | --- | --- | --- | --- |
|  |  | Heel strike | Toe-off | Calcaneus | Metatarsal head | Toe | Calcaneus | Metatarsal head | Toe |
| Slow | Control | 0.60[0.08] | 0.96[0.09] | 469[107] | 530[166] | 512[236] | 469[107] | 530[166] | 512[236] |
|  | SCO | 0.67[0.10] | 1.06[0.08] | 495[115] | 570[241] | 482[206] | 367[164] | 490[235] | 380[254] |
|  | LCO | 0.79[0.15] | 1.11[0.06] | 463[157] | 672[340] | 499[241] | 164[104] | 586[403] | 367[291] |
|  | SMHO | 0.57[0.06] | 0.95[0.12] | 512[115] | 562[151] | 535[218] | 405[97] | 437[189] | 346[249] |
|  | LMHO | 0.68[0.08] | 1.09[0.12] | 522[121] | 573[152] | 546[221] | 428[88] | 398[231] | 352[245] |
|  | LCOBS | 0.79[0.14] | 1.10[0.08] | 426[110] | 615[223] | 466[206] | 176[112] | 556[258] | 331[239] |
| Normal | Control | 0.61[0.11] | 0.96[0.12] | 490[117] | 535[165] | 681[331] | 490[117] | 535[165] | 681[331] |
|  | SCO | 0.69[0.10] | 1.09[0.07] | 551[134] | 559[175] | 613[209] | 405[196] | 492[110] | 432[219] |
|  | LCO | 0.79[0.12] | 1.11[0.09] | 566[178] | 654[201] | 585[247] | 146[44] | 602[180] | 413[288] |
|  | SMHO | 0.60[0.08] | 0.98[0.08] | 553[143] | 579[146] | 611[254] | 474[128] | 469[199] | 385[292] |
|  | LMHO | 0.70[0.16] | 1.05[0.06] | 557[154] | 722[289] | 691[271] | 454[129] | 484[260] | 418[266] |
|  | LCOBS | 0.74[0.11] | 1.09[0.10] | 541[174] | 724[299] | 627[240] | 149[65] | 652[326] | 350[322] |
| Fast | Control | 0.65[0.11] | 0.97[0.10] | 522[113] | 596[228] | 671[253] | 522[113] | 596[228] | 671[253] |
|  | SCO | 0.73[0.17] | 1.12[0.10] | 608[131] | 639[219] | 718[292] | 449[207] | 571[170] | 477[299] |
|  | LCO | 0.75[0.13] | 1.10[0.06] | 571[215] | 646[195] | 687[285] | 213[75] | 584[209] | 493[350] |
|  | SMHO | 0.62[0.11] | 0.98[0.13] | 603[145] | 652[155] | 693[256] | 535[156] | 519[179] | 477[294] |
|  | LMHO | 0.66[0.09] | 1.06[0.05] | 616[127] | 745[265] | 715[225] | 534[123] | 453[300] | 512[308] |
|  | LCOBS | 0.80[0.11] | 1.14[0.07] | 501[99] | 777[330] | 676[242] | 222[94] | 715[357] | 320[187] |

*APPP means average peak plantar pressure, SCO means small calcaneus offloading insole, LCO means larger calcaneus offloading insole, SMHO means small metatarsal head offloading insole, LMHO means large metatarsal head offloading insole, LCOBS means both sides large calcaneus offloading insole and std means standard deviation. “*

*“S3 Table. The percentage difference and P-value of average peak plantar pressure between five offloading conditions and the control condition at the foot region and region of interest for 10 subjects at three walking speeds.*

| Walking speed | Offloading condition | Velocity  (Control value, m/s;  *difference from control, % and [P-value]*) | | APPP at Region of Foot  (Control value, kPa;  *difference from control, % and [P-value]*) | | | APPP at Region of Interest  (Control value, kPa;  *difference from control, % and [P-value]*) | | |
| --- | --- | --- | --- | --- | --- | --- | --- | --- | --- |
|  |  | Heel strike | Toe-off | Calcaneus | Metatarsal head | Toe | Calcaneus | Metatarsal head | Toe |
| Slow | Control | 0.6 | 0.96 | 469 | 530 | 512 | 469 | 530 | 512 |
|  | SCO | 12[0.088] | 11[<0.05] | 6[0.175] | 4[0.234] | 9[0.673] | -23[<0.05] | -11[0.426] | -14[0.148] |
|  | LCO | 30[<0.05] | 17[<0.05] | -1[0.892] | 21[<0.05] | 14[0.752] | -62[<0.05] | -1[0.521] | -14[0.188] |
|  | SMHO | -6[0.123] | -2[0.603] | 11[0.189] | 8[0.265] | 24[0.73] | -13[<0.05] | -12[0.294] | -8[0.157] |
|  | LMHO | 15[<0.05] | 15[<0.05] | 13[0.096] | 10[0.24] | 24[0.665] | -7[0.121] | -21[0.195] | -10[0.17] |
|  | LCOBS | 29[<0.05] | 17[<0.05] | -7[0.277] | 16[0.031] | 6[0.535] | -59[<0.05] | 2[0.581] | -18[0.104] |
| Normal | Control | 0.61 | 0.96 | 490 | 535 | 681 | 490 | 535 | 681 |
|  | SCO | 15[<0.05] | 16[<0.05] | 13[<0.05] | 6[0.192] | -2[0.327] | -19[0.108] | -5[0.104] | -31[<0.05] |
|  | LCO | 32[<0.05] | 17[<0.05] | 17[0.142] | 26[<0.05] | -2[0.379] | -68[<0.05] | 16[<0.05] | -29[0.065] |
|  | SMHO | 1[0.917] | 3[0.391] | 15[0.149] | 12[0.158] | 0[0.256] | -3[0.58] | -7[0.429] | -32[0.067] |
|  | LMHO | 15[<0.05] | 11[<0.05] | 14[0.091] | 34[<0.05] | 21[0.809] | -8[0.192] | -7[0.579] | -18[0.113] |
|  | LCOBS | 25[<0.05] | 15[<0.05] | 14[0.423] | 36[<0.05] | 10[0.668] | -67[<0.05] | 18[0.11] | -33[0.073] |
| Fast | Control | 0.65 | 0.97 | 522 | 596 | 671 | 522 | 596 | 671 |
|  | SCO | 12[<0.05] | 16[<0.05] | 17[<0.05] | 9[0.146] | 9[0.366] | -14[0.264] | 0[0.674] | -25[0.085] |
|  | LCO | 23[<0.05] | 16[<0.05] | 7[<0.05] | 22[<0.05] | 3[0.851] | -57[<0.05] | 9[0.319] | -30[0.083] |
|  | SMHO | -1[0.726] | 2[0.487] | 17[<0.05] | 14[0.24] | 9[0.962] | 2[0.666] | -3[0.488] | -22[0.093] |
|  | LMHO | 1[0.944] | 11[0.053] | 19[<0.05] | 30[0.164] | 20[0.673] | 3[0.552] | -16[0.291] | -10[0.27] |
|  | LCOBS | 24[<0.05] | 19[<0.05] | 0[0.661] | 30[<0.05] | 13[0.967] | -55[<0.05] | 18[0.072] | -40[<0.05] |

*APPP means average peak plantar pressure, SCO means small calcaneus offloading insole, LCO means larger calcaneus offloading insole, SMHO means small metatarsal head offloading insole, LMHO means large metatarsal head offloading insole, LCOBS means both sides large calcaneus offloading insole and std means standard deviation. Positive value means increase and negative value means decrease.*”

**Comment 11:**

RESULTS: “Table 2 shows the mean pressure time integrals (PTI) percentage difference between five offloading conditions and control condition at RoF and RoI of 1o subjects at three walking speeds.”
Reviewer: It would be better to describe the mean and sd of each outcome and re analyze the statistical difference with the p-value among the 6 conditions of insole.
**Response:**

Thank you for your suggestions. We rebuild the table according to your advice in line 253 (page 11), line 267 (page 12) and line 438 (page 20) of our manuscript.

See the shaded sentences below for changes:

*“**Table 2 shows the mean pressure time integral at region of foot (RoF) and region of interest (RoI) (defined in Fig 3) and shank distal end velocity* *of five offloading conditions and the control condition for 10 subjects at three walking speeds. Percentage differences and P-values were calculated for the five offloading conditions and the control condition for 10 subjects at three walking speeds (S4 Table). “*

*“**Table 2. The mean pressure time integrals and shank distal end velocity of five offloading conditions and the control condition at the region of foot (RoF) and region of interest (RoI) for 10 subjects at three walking speeds.*

| Walking speed | Offloading condition | Velocity (m/s, *mean[std]*) | | PTI at RoF (kPa·s, *mean[std])* | | | PTI at RoI (kPa·s, *mean[std]*) | | |
| --- | --- | --- | --- | --- | --- | --- | --- | --- | --- |
|  |  | Heel strike | Toe-off | Calcaneus | Metatarsal head | Toe | Calcaneus | Metatarsal head | Toe |
| Slow | Control | 0.60[0.08] | 0.96[0.09] | 1726[293] | 1587[538] | 1009[668] | 1726[293] | 1587[538] | 1009[668] |
|  | SCO | 0.67[0.10] | 1.06[0.08] | 1781[340] | 1705[821] | 917[497] | 1171[533] | 1395[599] | 656[524] |
|  | LCO | 0.79[0.15] | 1.11[0.06] | 1628[534] | 1885[1121] | 897[495] | 406[179] | 1627[1323] | 528[475] |
|  | SMHO | 0.57[0.06] | 0.95[0.12] | 1938[310] | 1579[434] | 978[549] | 1548[306] | 1109[469] | 425[427] |
|  | LMHO | 0.68[0.08] | 1.09[0.12] | 1915[348] | 1542[480] | 1007[633] | 1622[266] | 1024[733] | 533[486] |
|  | LCOBS | 0.79[0.14] | 1.10[0.08] | 1547[400] | 1757[791] | 843[586] | 506[298] | 1483[797] | 428[316] |
| Normal | Control | 0.61[0.11] | 0.96[0.12] | 1457[197] | 1489[596] | 1402[982] | 1457[197] | 1489[596] | 1402[982] |
|  | SCO | 0.69[0.10] | 1.09[0.07] | 1648[359] | 1488[667] | 1090[618] | 1285[563] | 1182[368] | 574[326] |
|  | LCO | 0.79[0.12] | 1.11[0.09] | 1578[433] | 1553[448] | 996[493] | 475[285] | 1233[657] | 527[408] |
|  | SMHO | 0.60[0.08] | 0.98[0.08] | 1659[289] | 1501[524] | 883[298] | 1197[471] | 1100[737] | 440[336] |
|  | LMHO | 0.70[0.16] | 1.05[0.06] | 1574[368] | 1508[534] | 1071[507] | 1145[442] | 983[683] | 580[510] |
|  | LCOBS | 0.74[0.11] | 1.09[0.10] | 1513[501] | 1850[960] | 866[319] | 371[301] | 1585[954] | 430[341] |
| Fast | Control | 0.65[0.11] | 0.97[0.10] | 1352[217] | 1443[637] | 1094[501] | 1352[217] | 1443[637] | 1094[501] |
|  | SCO | 0.73[0.17] | 1.12[0.10] | 1530[305] | 1537[665] | 982[346] | 1147[476] | 1259[478] | 592[499] |
|  | LCO | 0.75[0.13] | 1.10[0.06] | 1356[435] | 1335[462] | 903[462] | 412[240] | 1127[439] | 622[416] |
|  | SMHO | 0.62[0.11] | 0.98[0.13] | 1530[243] | 1417[384] | 971[330] | 1210[235] | 1131[456] | 542[287] |
|  | LMHO | 0.66[0.09] | 1.06[0.05] | 1624[192] | 1449[315] | 1045[399] | 1306[172] | 934[699] | 687[462] |
|  | LCOBS | 0.80[0.11] | 1.14[0.07] | 1116[226] | 1691[723] | 978[471] | 475[326] | 1418[808] | 430[347] |

*PTI means pressure time integral, SCO means small calcaneus offloading insole, LCO means larger calcaneus offloading insole, SMHO means small metatarsal head offloading insole, LMHO means large metatarsal head offloading insole, LCOBS means both sides large calcaneus offloading insole, and std means standard deviation.***“**

*“S4 Table. The percentage difference and P-value of pressure time integral between five offloading conditions and the control condition at the foot region and region of interest for 10 subjects at three walking speeds.*

| Walking speed | Offloading condition | Velocity  (Control value, m/s;  *difference from control, % and [P-value]*) | | PTI at Region of Foot  (Control value, kPa·s;  *difference from control, % and [P-value]*) | | | PTI at Region of Interest  (Control value, kPa·s;  *difference from control, % and [P-value]*) | | |
| --- | --- | --- | --- | --- | --- | --- | --- | --- | --- |
|  |  | Heel strike | Toe-off | Calcaneus | Metatarsal head | Toe | Calcaneus | Metatarsal head | Toe |
| Slow | Control | 0.6 | 0.96 | 1726 | 1587 | 1009 | 1726 | 1587 | 1009 |
|  | SCO | 12[0.088] | 11[<0.05] | 4[0.58] | 4[0.68] | 15[0.48] | -29[<0.05] | -12[0.35] | -23[0.44] |
|  | LCO | 30[<0.05] | 17[<0.05] | -6[0.44] | 15[0.49] | 16[0.31] | -75[<0.05] | -4[0.35] | -29[0.53] |
|  | SMHO | -6[0.123] | -2[0.603] | 14[0.06] | 3[0.90] | 27[0.35] | -9[0.08] | -20[0.22] | -33[0.39] |
|  | LMHO | 15[<0.05] | 15[<0.05] | 12[0.10] | 3[0.95] | 26[0.34] | -3[0.38] | -26[0.26] | -23[0.74] |
|  | LCOBS | 29[<0.05] | 17[<0.05] | -9[0.15] | 15[0.62] | 0[0.87] | -68[<0.05] | -4[0.47] | -31[0.29] |
| Normal | Control | 0.61 | 0.96 | 1457 | 1489 | 1402 | 1457 | 1489 | 1402 |
|  | SCO | 15[<0.05] | 16[<0.05] | 12[<0.05] | -0[0.99] | -7[0.49] | -12[0.32] | -15[<0.05] | -45[<0.05] |
|  | LCO | 32[<0.05] | 17[<0.05] | 7[0.30] | 11[0.54] | -5[0.68] | -65[<0.05] | -17[0.11] | -41[0.07] |
|  | SMHO | 1[0.917] | 3[0.391] | 14[0.06] | 8[0.95] | -12[0.46] | -19[<0.05] | -18[0.23] | -50[<0.05] |
|  | LMHO | 15[<0.05] | 11[<0.05] | 8[0.28] | 6[0.92] | 12[0.97] | -22[<0.05] | -32[0.09] | -29[0.14] |
|  | LCOBS | 25[<0.05] | 15[<0.05] | 4[0.73] | 28[0.16] | -11[0.47] | -73[<0.05] | 4[0.63] | -49[<0.05] |
| Fast | Control | 0.65 | 0.97 | 1352 | 1443 | 1094 | 1352 | 1443 | 1094 |
|  | SCO | 12[<0.05] | 16[<0.05] | 14[0.05] | 7[NA] | 1[0.36] | -16[0.18] | -8[0.97] | -42[<0.05] |
|  | LCO | 23[<0.05] | 16[<0.05] | -1[0.93] | 6[NA] | -11[0.22] | -69[0.39] | -10[0.74] | -33[0.37] |
|  | SMHO | -1[0.726] | 2[0.487] | 15[0.06] | 8[NA] | 8[0.51] | -9[0.08] | -7[0.11] | -34[<0.05] |
|  | LMHO | 1[0.944] | 11[0.053] | 22[<0.05] | 12[NA] | 20[0.83] | -1[0.54] | -27[0.74] | -16[0.14] |
|  | LCOBS | 24[<0.05] | 19[<0.05] | -13[0.08] | 24[NA] | 8[0.60] | -65[<0.05] | -1[0.86] | -45[<0.05] |

*PTI means pressure time integral, SCO means small calcaneus offloading insole, LCO means larger calcaneus offloading insole, SMHO means small metatarsal head offloading insole, LMHO means large metatarsal head offloading insole, LCOBS means both sides large calcaneus offloading insole, and std means standard deviation. The positive percentage means increase, and the negative percentage means decrease. Positive value means increase and negative value means decrease.* **“**

**Comment 12:**

RESULTS: “Table 3 shows the mean value of width of centre of force (CoF), length of CoF, and asymmetry difference between five offloading conditions and control condition of 10 subjects at three walking speeds.”
Reviewer: It would be better to describe the mean and sd of each outcome and re analyze the statistical difference with the p-value among the 6 conditions of insole.
**Response:**

Thank you for your suggestions. We rebuild the table according to your advice in line 2815 (page 13), line 287 (page 13) and line 444 (page 20) of our manuscript.

See the shaded sentences below for changes:

***“****Table 3 shows the mean value of width of centre of force (CoF), length of CoF, and asymmetry of width and length of five offloading conditions and control condition of 10 subjects at three walking speeds. Percentage differences and P-values were calculated for the five offloading conditions and the control condition for 10 subjects at three walking speeds (S5 Table).* ***“***

*“Table 3. The mean centre of force width and length, and asymmetry of width and length of five offloading conditions and the control condition for 10 subjects at three walking speeds.*

| Mean and standard deviation data | | | | | |
| --- | --- | --- | --- | --- | --- |
| Walking speed | Offloading condition | Width (m, *mean[std]*) | Length (m, *mean[std]*) | Asymmetry of width (*mean[std]*) | Asymmetry of length (*mean[std]*) |
| Slow | Control | 0.21[0.04] | 0.31[0.05] | 5.90[4.52] | 11.33[4.70] |
|  | SCO | 0.21[0.03] | 0.32[0.04] | 4.60[0.68] | 13.70[7.27] |
|  | LCO | 0.22[0.04] | 0.33[0.05] | 5.41[2.11] | 12.29[4.16] |
|  | SMHO | 0.21[0.04] | 0.33[0.05] | 4.75[2.10] | 11.07[4.23] |
|  | LMHO | 0.21[0.04] | 0.33[0.06] | 4.85[1.58] | 13.06[5.05] |
|  | LCOBS | 0.21[0.04] | 0.33[0.05] | 5.42[2.09] | 12.69[2.74] |
| Normal | Control | 0.22[0.04] | 0.35[0.06] | 4.81[1.26] | 9.15[2.60] |
|  | SCO | 0.22[0.04] | 0.36[0.06] | 4.84[1.66] | 9.10[3.55] |
|  | LCO | 0.22[0.04] | 0.35[0.06] | 5.07[2.08] | 7.38[2.23] |
|  | SMHO | 0.22[0.03] | 0.37[0.05] | 5.70[2.10] | 9.03[3.21] |
|  | LMHO | 0.22[0.03] | 0.37[0.06] | 6.35[2.34] | 8.90[4.65] |
|  | LCOBS | 0.21[0.04] | 0.36[0.06] | 4.63[1.27] | 8.90[3.14] |
| Fast | Control | 0.21[0.03] | 0.41[0.06] | 5.08[1.65] | 7.18[1.91] |
|  | SCO | 0.21[0.02] | 0.41[0.06] | 5.78[1.84] | 8.69[2.44] |
|  | LCO | 0.22[0.03] | 0.42[0.06] | 6.96[4.32] | 8.33[4.61] |
|  | SMHO | 0.21[0.03] | 0.42[0.07] | 6.23[3.81] | 8.03[3.10] |
|  | LMHO | 0.21[0.04] | 0.43[0.06] | 7.07[2.14] | 8.39[3.28] |
|  | LCOBS | 0.22[0.03] | 0.42[0.05] | 6.04[1.51] | 8.13[4.77] |

*Control means control flat insole, SCO means small calcaneus offloading insole, LCO means larger calcaneus offloading insole, SMHO means small metatarsal head offloading insole, LMHO means large metatarsal head offloading insole, LCOBS means both sides large calcaneus offloading insole, and std means standard deviation.* **“**

*“S5 Table. The mean centre of force width and length along with the percentage difference and P-value, and asymmetry of width and length along with the difference and P-value between five offloading conditions and the control condition for 10 subjects at three walking speeds.*

|  |  | Percentage difference with the P-value | | Difference with the P-value | |
| --- | --- | --- | --- | --- | --- |
| Walking speed | Offloading condition | Width (Control value, m;  *difference from control, % and [P-value]*) | Length (Control value, m;  *difference from control, % and [P-value]*) | Asymmetry of width (Control value, %;  *difference from control, % and [P-value]*) | Asymmetry of length  (Control value, %;  *difference from control, % and [P-value]*) |
| Slow | Control | 0.21 | 0.31 | 5.90 | 11.33 |
|  | SCO | 4[1.00] | 2[0.64] | 0.08[0.44] | 0.30[0.37] |
|  | LCO | 7[0.54] | 7[0.06] | 0.86[0.54] | 0.80[0.73] |
|  | SMHO | 5[0.62] | 3[0.27] | 0.30[0.27] | -0.52[0.75] |
|  | LMHO | 6[0.53] | 4[0.16] | 0.50[0.53] | 2.38[0.27] |
|  | LCOBS | 5[0.68] | 7[<0.05] | 0.77[0.66] | 1.99[0.29] |
| Normal | Control | 0.22 | 0.35 | 4.81 | 9.15 |
|  | SCO | 1[1.00] | 1[0.42] | -0.08[0.92] | -0.05[0.96] |
|  | LCO | 5[0.20] | 1[0.56] | 0.26[0.59] | -1.77[0.11] |
|  | SMHO | 1[0.70] | 6[<0.05] | 0.89[0.09] | -0.12[0.91] |
|  | LMHO | 1[0.75] | 4[<0.05] | 1.54[<0.05] | -0.24[0.88] |
|  | LCOBS | -1[0.66] | 4[<0.05] | -0.18[0.71] | -0.25[0.82] |
| Fast | Control | 0.21 | 0.41 | 5.08 | 7.18 |
|  | SCO | -3[0.28] | 1[0.67] | 0.97[0.28] | 1.50[<0.05] |
|  | LCO | -1[0.65] | 5[<0.05] | 1.55[0.25] | 1.12[0.36] |
|  | SMHO | 0[0.85] | 3[0.38] | 1.30[0.33] | 0.75[0.41] |
|  | LMHO | -1[0.33] | 6[<0.05] | 2.10[<0.05] | 1.39[0.22] |
|  | LCOBS | 1[0.74] | 3[0.09] | 0.96[<0.05] | 0.95[0.46] |

*Control means control flat insole, SCO means small calcaneus offloading insole, LCO means larger calcaneus offloading insole, SMHO means small metatarsal head offloading insole, LMHO means large metatarsal head offloading insole, LCOBS means both sides large calcaneus offloading insole, and std means standard deviation. Positive valve means increase and negative value means decrease.* **“**

**Comment 13:**
DISCUSSION: “A possible way to solve this problem could be to modify the insole to minimise pressure in the aperture region rather than just over the average peak plantar pressure for metatarsal head or calcaneus.”
Reviewer: The author should clarify how the present study design the insole to solve the problem from the previous studies.
**Response:**

Thank you for pointing this out. Our study was not designed to be an intervention to solve the problem of DFU. Rather it aimed to investigate the relationship between offloading regions in the insole and their effect on both the plantar pressure and the gait kinematics. We clarify this in line 78 (page 4) of our manuscript.

We recognise that the aim of the study may not have articulated this clearly, so we have reworded this to try to improve our communication.

See the shaded sentences below for changes:

*“This study aims to investigate the effect of offloading insoles on plantar pressure and their influence on the gait kinematics. To the authors knowledge this is the first study which jointly measures both plantar pressure and gait kinematics using offloading insoles. The results should help to guide the future development and evaluation of offloading insoles that could reduce the risk of high plantar pressure and altered gait styles in a diabetic population. This is a pilot study and therefore only aims to measure the effects of the insole intervention rather than to solve any issues observed. The objectives of this study are: (a) evaluating the offloading insoles effect on high risk region; (b) estimating the offloading effect on gait kinematics and kinetics; (c) examining the subjects gait asymmetry change; (d) and analysing the relationship between the gait kinematics, kinetics and plantar pressure.”*

**Comment 14:**

DISCUSSION: “No significant trend is found about the sagittal angles but the heel strike and toe-off velocity increase between 1% and 32% respectively when applying offloading insoles except SMHO, which is not reported in other papers to the authors’ knowledge.”
Reviewer: Please describe more detail about how to receive the significant value, since the author did not report the significant value in the table data.
**Response:**

Thank you for pointing this out. We apologise for the incorrect use of the term in this study. The word 'significant' has been replaced to accurately reflect that this pilot study does not report statistically significant data. In addition, we have added the statistical method section and clarified the limitation of the small sample size in our manuscript.

See the shaded sentences below for changes:

Line 273 (page 13):

*“Offloading insoles except SMHO increased the heel strike velocity by a mean of 19% (range 1% to 32%). Toe-off velocity was observed to increase by a mean of 15% (range 11% to 19%) when applying offloading insoles except SMHO. LCO increased the heel strike velocity and toe off velocity by a mean of 23% (range 16% to 32%, P<0.05)* during walking at three speeds*.* *LMHO also observed increases in heel strike velocity and toe off velocity during slow and normal walking (P <0.05). No difference in the right thigh, shank and foot angle was observed*.*”*

Line 302 (page 15):

*“However, it is important to note that this trend was not observed during fast walking.”*

Line 304 (page 15):

*“…This trend is not observed when walking at normal and fast speeds… “*

Line 326 (page 16):

*…No* *difference is observed about the sagittal angles but the heel strike and toe-off velocity increase between 1% and 32% respectively when applying…*

Line 342 (page 16):

*“…Consequently, the centre of force figure is important because it can show the change in gait kinematics and… “*

Additionally, we added comments on the statistical methods – see comment/response 9.

And we also added the limitation of the small sample size Line 353 (page 17):

*“Our sample size was small and chosen for convenience sampling, as is common for pilot studies of this type [20] therefore our results may not be statistically significant. However, the results of this study do show some indicative relationships between gait kinematics, and plantar pressure for offloading insole conditions but further work would be needed to determine if these relationships are significant. This study involved healthy participants to assess insole design safely and effect on gait kinematics and plantar pressure. This information could then be used in future studies on diabetic populations to ensure a safe and non-harmful approach.* *Although the current findings do not directly apply to diabetic foot ulcer management or diabetic subjects, they are indicative of general gait changes which will help in guiding the development and evaluation of insoles that could significantly reduce the risk of diabetic foot ulceration.”*

**Comment 15:**

DISCUSSION: “Although as a pilot study, the result of this study is not statistically significant, it gives a general trend of relationship between gait kinematics, kinetics, asymmetry and plantar pressure.”
Reviewer: The author should add in the title about the word “Pilot study”. Again, please describe more detail about how to receive the significant value, since the author did not report the significant value in the table data.

**Response:**

Thank you for point this out. We modify the title in line 1 (page 1) of our manuscript.

See the shaded sentences below for changes:

“The effect of calcaneus and metatarsal head offloading insoles on healthy subjects’ gait kinematics, kinetics, asymmetry, and the implications for plantar pressure management: a pilot study.”

Please also see response to comment 14 above.

Thank you again to the reviewers and the editorial team. We feel their suggestions and the changes we have made to the manuscript have improved the clarity of the paper for the reader. We hope that it will now be possible for you to be able to consider this work for publication in your journal. Thank you in advance for your consideration and time to review this.

Kind regards

Jiwei Shuang
