# Supplementary material for: The effect of calcaneus and metatarsal head offloading insoles on healthy subjects’ gait kinematics, kinetics, asymmetry, and the implications for plantar pressure management: A pilot study
Source: PLoS One. 2024 May 17;19(5):e0303826. doi: 10.1371/journal.pone.0303826 (PMC11101073; doi:10.1371/journal.pone.0303826)
Supplement: Attachment — Submitted filename: Response to Reviewers.docx [file pone.0303826.s004.docx]

School of Engineering

The University of Manchester

Engineering Building A

Manchester M13 9PL

+44(0)161 529 3401

[Jiawei.shuang@manchester.ac.uk](mailto:Jiawei.shuang@manchester.ac.uk)

[www.mace.manchester.ac.uk](http://www.mace.manchester.ac.uk)

10^th^ April 2024

Dear Dr Surapong Chatpun,

**Re: PLOS ONE Decision: Revision required [PONE-D-23-30055] - [EMID:db690a3d5d331f19]**

Thank you for giving us the opportunity to submit a revised draft of our manuscript titled ‘**The effect of calcaneus and metatarsal head offloading insoles on healthy subjects’ gait kinematics, kinetics, asymmetry, and the implications for plantar pressure management**’ to PLOS ONE.

We appreciate the time and effort that you and the reviewers have dedicated to providing your valuable feedback and comments on our manuscript. We have tried to incorporate changes to reflect the suggestions provided by the reviewers.

Here is the response to the reviewer’s comments and concerns. Lines quoted are for the ‘Revised Manuscript with Track Changes’.

**Comments from the Review #1:**

**Review Comments:**

The authors addressed the responses to the comments completely and clearly. The authors added more information to make the content better than the previous version.

**Response:**

Thank you for your positive feedback. We're glad to hear that the additional information and clarifications have enhanced the content and addressed your comments effectively. If there are any more questions or further input, we'd be happy to hear them.

**Comments from the Review #2:**

**Review Comments:**

My major concern is still the statistical analysis of this study, the author should clearly present the real number of p-value in the table, the writing only P < 0.05 within the text is not enough. Please re-consider to present the p-value in the table other than only the mean and standard deviation and rewrite the text within the paragraph especially the variables without significant and the variables with significance.

**Response:**

Thank you for your comment. We appreciate your desire to include all the P-values within the main text. We have now moved this information from the supplementary information to the main text and made the following modifications:

- Added P-values to table 1-4.
- Added P-values to the text descriptions explaining if they were significant or insignificant.

Full details of this are shown below in the “Changes Section” and in the amended manuscript attached.

Thank you again for your time and consideration of our research paper.

Kind regards

Jiawei Shuang

**Changes Section**

**Please see the tables below for changes associated with P-value real number:**

**Table 1. The mean average peak plantar pressure and shank distal end velocity of five offloading conditions and the control condition at the region of foot (RoF) and region of interest (RoI) for 10 subjects at three walking speeds.**

| Walking speed | Offloading condition | Velocity (m/s, mean[std]) | | APPP at region of foot (kPa, mean[std]) | | | APPP at region of interest (kPa, mean[std]) | | |
| --- | --- | --- | --- | --- | --- | --- | --- | --- | --- |
|  |  | Heel strike | Toe-off | Calcaneus | Metatarsal head | Toe | Calcaneus | Metatarsal head | Toe |
| Slow | Control | 0.60[0.08] | 0.96[0.09] | 469[107] | 530[166] | 512[236] | 469[107] | 530[166] | 512[236] |
|  | SCO | 0.67[0.10] | 1.06[0.08] | 495[115] | 570[241] | 482[206] | 367[164] | 490[235] | 380[254] |
|  | LCO | 0.79[0.15] | 1.11[0.06] | 463[157] | 672[340] | 499[241] | 164[104] | 586[403] | 367[291] |
|  | SMHO | 0.57[0.06] | 0.95[0.12] | 512[115] | 562[151] | 535[218] | 405[97] | 437[189] | 346[249] |
|  | LMHO | 0.68[0.08] | 1.09[0.12] | 522[121] | 573[152] | 546[221] | 428[88] | 398[231] | 352[245] |
|  | LCOBS | 0.79[0.14] | 1.10[0.08] | 426[110] | 615[223] | 466[206] | 176[112] | 556[258] | 331[239] |
| Normal | Control | 0.61[0.11] | 0.96[0.12] | 490[117] | 535[165] | 681[331] | 490[117] | 535[165] | 681[331] |
|  | SCO | 0.69[0.10] | 1.09[0.07] | 551[134] | 559[175] | 613[209] | 405[196] | 492[110] | 432[219] |
|  | LCO | 0.79[0.12] | 1.11[0.09] | 566[178] | 654[201] | 585[247] | 146[44] | 602[180] | 413[288] |
|  | SMHO | 0.60[0.08] | 0.98[0.08] | 553[143] | 579[146] | 611[254] | 474[128] | 469[199] | 385[292] |
|  | LMHO | 0.70[0.16] | 1.05[0.06] | 557[154] | 722[289] | 691[271] | 454[129] | 484[260] | 418[266] |
|  | LCOBS | 0.74[0.11] | 1.09[0.10] | 541[174] | 724[299] | 627[240] | 149[65] | 652[326] | 350[322] |
| Fast | Control | 0.65[0.11] | 0.97[0.10] | 522[113] | 596[228] | 671[253] | 522[113] | 596[228] | 671[253] |
|  | SCO | 0.73[0.17] | 1.12[0.10] | 608[131] | 639[219] | 718[292] | 449[207] | 571[170] | 477[299] |
|  | LCO | 0.75[0.13] | 1.10[0.06] | 571[215] | 646[195] | 687[285] | 213[75] | 584[209] | 493[350] |
|  | SMHO | 0.62[0.11] | 0.98[0.13] | 603[145] | 652[155] | 693[256] | 535[156] | 519[179] | 477[294] |
|  | LMHO | 0.66[0.09] | 1.06[0.05] | 616[127] | 745[265] | 715[225] | 534[123] | 453[300] | 512[308] |
|  | LCOBS | 0.80[0.11] | 1.14[0.07] | 501[99] | 777[330] | 676[242] | 222[94] | 715[357] | 320[187] |
| Walking speed | Offloading condition | Velocity  (Control value, m/s;  *difference from control, % and [P-value]*) | | APPP at region of foot  (Control value, kPa;  *difference from control, % and [P-value]*) | | | APPP at region of interest  (Control value, kPa;  *difference from control, % and [P-value]*) | | |
|  |  | Heel strike | Toe-off | Calcaneus | Metatarsal head | Toe | Calcaneus | Metatarsal head | Toe |
| Slow | Control | 0.6 | 0.96 | 469 | 530 | 512 | 469 | 530 | 512 |
|  | SCO | 12[0.091] | 11[0.000] | 6[0.175] | 4[0.234] | 9[0.673] | -23[0.040] | -11[0.426] | -14[0.148] |
|  | LCO | 30[0.003] | 17[0.001] | -1[0.892] | 21[0.049] | 14[0.752] | -62[0.001] | -1[0.521] | -14[0.188] |
|  | SMHO | -6[0.117] | -2[0.598] | 11[0.189] | 8[0.265] | 24[0.730] | -13[0.006] | -12[0.294] | -8[0.157] |
|  | LMHO | 15[0.020] | 15[0.044] | 13[0.096] | 10[0.240] | 24[0.665] | -7[0.121] | -21[0.195] | -10[0.170] |
|  | LCOBS | 29[0.004] | 17[0.010] | -7[0.277] | 16[0.031] | 6[0.535] | -59[0.001] | 2[0.581] | -18[0.104] |
| Normal | Control | 0.61 | 0.96 | 490 | 535 | 681 | 490 | 535 | 681 |
|  | SCO | 15[0.005] | 16[0.001] | 13[0.002] | 6[0.192] | -2[0.327] | -19[0.108] | -5[0.104] | -31[0.030] |
|  | LCO | 32[0.002] | 17[0.004] | 17[0.142] | 26[0.014] | -2[0.379] | -68[0.001] | 16[0.039] | -29[0.065] |
|  | SMHO | 1[0.909] | 3[0.398] | 15[0.149] | 12[0.158] | 0[0.256] | -3[0.580] | -7[0.429] | -32[0.067] |
|  | LMHO | 15[0.004] | 11[0.006] | 14[0.091] | 34[0.028] | 21[0.809] | -8[0.192] | -7[0.579] | -18[0.113] |
|  | LCOBS | 25[0.008] | 15[0.013] | 14[0.423] | 36[0.015] | 10[0.668] | -67[0.001] | 18[0.11] | -33[0.073] |
| Fast | Control | 0.65 | 0.97 | 522 | 596 | 671 | 522 | 596 | 671 |
|  | SCO | 12[0.006] | 16[0.004] | 17[0.001] | 9[0.146] | 9[0.366] | -14[0.264] | 0[0.674] | -25[0.085] |
|  | LCO | 23[0.026] | 16[0.005] | 7[0.485] | 22[0.025] | 3[0.851] | -57[0.001] | 9[0.319] | -30[0.083] |
|  | SMHO | -1[0.755] | 2[0.426] | 17[0.038] | 14[0.240] | 9[0.962] | 2[0.666] | -3[0.488] | -22[0.093] |
|  | LMHO | 1[0.945] | 11[0.023] | 19[0.004] | 30[0.164] | 20[0.673] | 3[0.552] | -16[0.291] | -10[0.270] |
|  | LCOBS | 24[0.001] | 19[0.002] | 0[0.661] | 30[0.002] | 13[0.967] | -55[0.001] | 18[0.072] | -40[0.020] |

Percentage differences and P-values were calculated for the five offloading conditions and the control condition for 10 subjects at three walking speeds. P-values smaller than 0.05 were underlined. APPP means average peak plantar pressure, SCO means small calcaneus offloading insole, LCO means larger calcaneus offloading insole, SMHO means small metatarsal head offloading insole, LMHO means large metatarsal head offloading insole, LCOBS means both sides large calcaneus offloading insole and std means standard deviation.

**Table 2. The mean pressure time integrals and shank distal end velocity of five offloading conditions and the control condition at the region of foot (RoF) and region of interest (RoI) for 10 subjects at three walking speeds.**

| Walking speed | Offloading condition | Velocity (m/s, mean[std]) | | PTI at region of foot (kPa·s, mean[std]) | | | PTI at region of interest (kPa·s, mean[std]) | | |
| --- | --- | --- | --- | --- | --- | --- | --- | --- | --- |
|  |  | Heel strike | Toe-off | Calcaneus | Metatarsal head | Toe | Calcaneus | Metatarsal head | Toe |
| Slow | Control | 0.60[0.08] | 0.96[0.09] | 1726[293] | 1587[538] | 1009[668] | 1726[293] | 1587[538] | 1009[668] |
|  | SCO | 0.67[0.10] | 1.06[0.08] | 1781[340] | 1705[821] | 917[497] | 1171[533] | 1395[599] | 656[524] |
|  | LCO | 0.79[0.15] | 1.11[0.06] | 1628[534] | 1885[1121] | 897[495] | 406[179] | 1627[1323] | 528[475] |
|  | SMHO | 0.57[0.06] | 0.95[0.12] | 1938[310] | 1579[434] | 978[549] | 1548[306] | 1109[469] | 425[427] |
|  | LMHO | 0.68[0.08] | 1.09[0.12] | 1915[348] | 1542[480] | 1007[633] | 1622[266] | 1024[733] | 533[486] |
|  | LCOBS | 0.79[0.14] | 1.10[0.08] | 1547[400] | 1757[791] | 843[586] | 506[298] | 1483[797] | 428[316] |
| Normal | Control | 0.61[0.11] | 0.96[0.12] | 1457[197] | 1489[596] | 1402[982] | 1457[197] | 1489[596] | 1402[982] |
|  | SCO | 0.69[0.10] | 1.09[0.07] | 1648[359] | 1488[667] | 1090[618] | 1285[563] | 1182[368] | 574[326] |
|  | LCO | 0.79[0.12] | 1.11[0.09] | 1578[433] | 1553[448] | 996[493] | 475[285] | 1233[657] | 527[408] |
|  | SMHO | 0.60[0.08] | 0.98[0.08] | 1659[289] | 1501[524] | 883[298] | 1197[471] | 1100[737] | 440[336] |
|  | LMHO | 0.70[0.16] | 1.05[0.06] | 1574[368] | 1508[534] | 1071[507] | 1145[442] | 983[683] | 580[510] |
|  | LCOBS | 0.74[0.11] | 1.09[0.10] | 1513[501] | 1850[960] | 866[319] | 371[301] | 1585[954] | 430[341] |
| Fast | Control | 0.65[0.11] | 0.97[0.10] | 1352[217] | 1443[637] | 1094[501] | 1352[217] | 1443[637] | 1094[501] |
|  | SCO | 0.73[0.17] | 1.12[0.10] | 1530[305] | 1537[665] | 982[346] | 1147[476] | 1259[478] | 592[499] |
|  | LCO | 0.75[0.13] | 1.10[0.06] | 1356[435] | 1335[462] | 903[462] | 412[240] | 1127[439] | 622[416] |
|  | SMHO | 0.62[0.11] | 0.98[0.13] | 1530[243] | 1417[384] | 971[330] | 1210[235] | 1131[456] | 542[287] |
|  | LMHO | 0.66[0.09] | 1.06[0.05] | 1624[192] | 1449[315] | 1045[399] | 1306[172] | 934[699] | 687[462] |
|  | LCOBS | 0.80[0.11] | 1.14[0.07] | 1116[226] | 1691[723] | 978[471] | 475[326] | 1418[808] | 430[347] |
| Walking speed | Offloading condition | Velocity  (Control value, m/s;  *difference from control, % and [P-value]*) | | PTI at region of foot  (Control value, kPa·s;  *difference from control, % and [P-value]*) | | | PTI at region of interest  (Control value, kPa·s;  *difference from control, % and [P-value]*) | | |
|  |  | Heel strike | Toe-off | Calcaneus | Metatarsal head | Toe | Calcaneus | Metatarsal head | Toe |
| Slow | Control | 0.6 | 0.96 | 1726 | 1587 | 1009 | 1726 | 1587 | 1009 |
|  | SCO | 12[0.091] | 11[0.000] | 4[0.581] | 4[0.679] | 15[0.480] | -29[0.025] | -12[0.345] | -23[0.442] |
|  | LCO | 30[0.003] | 17[0.001] | -6[0.437] | 15[0.489] | 16[0.314] | -75[0.000] | -4[0.347] | -29[0.532] |
|  | SMHO | -6[0.117] | -2[0.598] | 14[0.061] | 3[0.899] | 27[0.354] | -9[0.082] | -20[0.218] | -33[0.394] |
|  | LMHO | 15[0.020] | 15[0.044] | 12[0.104] | 3[0.952] | 26[0.338] | -3[0.379] | -26[0.259] | -23[0.743] |
|  | LCOBS | 29[0.004] | 17[0.010] | -9[0.151] | 15[0.616] | 0[0.867] | -68[0.000] | -4[0.471] | -31[0.291] |
| Normal | Control | 0.61 | 0.96 | 1457 | 1489 | 1402 | 1457 | 1489 | 1402 |
|  | SCO | 15[0.005] | 16[0.001] | 12[0.039] | -0[0.993] | -7[0.487] | -12[0.321] | -15[0.046] | -45[0.040] |
|  | LCO | 32[0.002] | 17[0.004] | 7[0.301] | 11[0.540] | -5[0.682] | -65[0.000] | -17[0.111] | -41[0.074] |
|  | SMHO | 1[0.909] | 3[0.398] | 14[0.055] | 8[0.950] | -12[0.460] | -19[0.054] | -18[0.232] | -50[0.039] |
|  | LMHO | 15[0.004] | 11[0.006] | 8[0.278] | 6[0.916] | 12[0.970] | -22[0.032] | -32[0.091] | -29[0.141] |
|  | LCOBS | 25[0.008] | 15[0.013] | 4[0.731] | 28[0.158] | -11[0.469] | -73[0.000] | 4[0.634] | -49[0.042] |
| Fast | Control | 0.65 | 0.97 | 1352 | 1443 | 1094 | 1352 | 1443 | 1094 |
|  | SCO | 12[0.006] | 16[0.004] | 14[0.051] | 7[NA] | 1[0.357] | -16[0.185] | -8[0.970] | -42[0.017] |
|  | LCO | 23[0.026] | 16[0.005] | -1[0.925] | 6[NA] | -11[0.222] | -69[0.389] | -10[0.742] | -33[0.369] |
|  | SMHO | -1[0.755] | 2[0.426] | 15[0.063] | 8[NA] | 8[0.506] | -9[0.081] | -7[0.113] | -34[0.033] |
|  | LMHO | 1[0.945] | 11[0.023] | 22[0.002] | 12[NA] | 20[0.825] | -1[0.537] | -27[0.738] | -16[0.138] |
|  | LCOBS | 24[0.001] | 19[0.002] | -13[0.078] | 24[NA] | 8[0.598] | -65[0.000] | -1[0.864] | -45[0.017] |

Percentage differences and P-values were calculated for the five offloading conditions and the control condition for 10 subjects at three walking speeds. P-values smaller than 0.05 were underlined. PTI means pressure time integral, SCO means small calcaneus offloading insole, LCO means larger calcaneus offloading insole, SMHO means small metatarsal head offloading insole, LMHO means large metatarsal head offloading insole, LCOBS means both sides large calcaneus offloading insole, and std means standard deviation.

**Table 3. The mean centre of force width and length, and asymmetry of width and length of five offloading conditions and the control condition for 10 subjects at three walking speeds.**

| Mean and standard deviation data | | | | | |
| --- | --- | --- | --- | --- | --- |
| Walking speed | Offloading condition | Width (m, mean[std]) | Length (m, mean[std]) | Asymmetry of width (mean[std]) | Asymmetry of length (mean[std]) |
| Slow | Control | 0.21[0.04] | 0.31[0.05] | 5.90[4.52] | 11.33[4.70] |
|  | SCO | 0.21[0.03] | 0.32[0.04] | 4.60[0.68] | 13.70[7.27] |
|  | LCO | 0.22[0.04] | 0.33[0.05] | 5.41[2.11] | 12.29[4.16] |
|  | SMHO | 0.21[0.04] | 0.33[0.05] | 4.75[2.10] | 11.07[4.23] |
|  | LMHO | 0.21[0.04] | 0.33[0.06] | 4.85[1.58] | 13.06[5.05] |
|  | LCOBS | 0.21[0.04] | 0.33[0.05] | 5.42[2.09] | 12.69[2.74] |
| Normal | Control | 0.22[0.04] | 0.35[0.06] | 4.81[1.26] | 9.15[2.60] |
|  | SCO | 0.22[0.04] | 0.36[0.06] | 4.84[1.66] | 9.10[3.55] |
|  | LCO | 0.22[0.04] | 0.35[0.06] | 5.07[2.08] | 7.38[2.23] |
|  | SMHO | 0.22[0.03] | 0.37[0.05] | 5.70[2.10] | 9.03[3.21] |
|  | LMHO | 0.22[0.03] | 0.37[0.06] | 6.35[2.34] | 8.90[4.65] |
|  | LCOBS | 0.21[0.04] | 0.36[0.06] | 4.63[1.27] | 8.90[3.14] |
| Fast | Control | 0.21[0.03] | 0.41[0.06] | 5.08[1.65] | 7.18[1.91] |
|  | SCO | 0.21[0.02] | 0.41[0.06] | 5.78[1.84] | 8.69[2.44] |
|  | LCO | 0.22[0.03] | 0.42[0.06] | 6.96[4.32] | 8.33[4.61] |
|  | SMHO | 0.21[0.03] | 0.42[0.07] | 6.23[3.81] | 8.03[3.10] |
|  | LMHO | 0.21[0.04] | 0.43[0.06] | 7.07[2.14] | 8.39[3.28] |
|  | LCOBS | 0.22[0.03] | 0.42[0.05] | 6.04[1.51] | 8.13[4.77] |
|  |  | Percentage difference with the P-value | | Difference with the P-value | |
| Walking speed | Offloading condition | Width (Control value, m;  *difference from control, % and [P-value]*) | Length (Control value, m;  *difference from control, % and [P-value]*) | Asymmetry of width (Control value, %;  *difference from control, % and [P-value]*) | Asymmetry of length  (Control value, %;  *difference from control, % and [P-value]*) |
| Slow | Control | 0.21 | 0.31 | 5.90 | 11.33 |
|  | SCO | 4[1.000] | 2[0.641] | 0.08[0.439] | 0.30[0.367] |
|  | LCO | 7[0.537] | 7[0.062] | 0.86[0.543] | 0.80[0.731] |
|  | SMHO | 5[0.625] | 3[0.272] | 0.30[0.266] | -0.52[0.748] |
|  | LMHO | 6[0.534] | 4[0.155] | 0.50[0.534] | 2.38[0.266] |
|  | LCOBS | 5[0.681] | 7[0.051] | 0.77[0.664] | 1.99[0.286] |
| Normal | Control | 0.22 | 0.35 | 4.81 | 9.15 |
|  | SCO | 1[1.000] | 1[0.423] | -0.08[0.920] | -0.05[0.961] |
|  | LCO | 5[0.204] | 1[0.560] | 0.26[0.590] | -1.77[0.105] |
|  | SMHO | 1[0.695] | 6[0.049] | 0.89[0.087] | -0.12[0.911] |
|  | LMHO | 1[0.746] | 4[0.018] | 1.54[0.019] | -0.24[0.880] |
|  | LCOBS | -1[0.656] | 4[0.022] | -0.18[0.709] | -0.25[0.824] |
| Fast | Control | 0.21 | 0.41 | 5.08 | 7.18 |
|  | SCO | -3[0.285] | 1[0.674] | 0.97[0.277] | 1.50[0.048] |
|  | LCO | -1[0.649] | 5[0.022] | 1.55[0.252] | 1.12[0.364] |
|  | SMHO | 0[0.853] | 3[0.378] | 1.30[0.331] | 0.75[0.411] |
|  | LMHO | -1[0.329] | 6[0.014] | 2.10[0.022] | 1.39[0.223] |
|  | LCOBS | 1[0.743] | 3[0.090] | 0.96[0.041] | 0.95[0.458] |

Percentage differences and P-values were calculated for the five offloading conditions and the control condition for 10 subjects at three walking speeds. P-values smaller than 0.05 were underlined. Control means control flat insole, SCO means small calcaneus offloading insole, LCO means larger calcaneus offloading insole, SMHO means small metatarsal head offloading insole, LMHO means large metatarsal head offloading insole, LCOBS means both sides large calcaneus offloading insole, and std means standard deviation.

**Table 4. The mean arch index and total contact area of five offloading conditions and the control condition for 10 subjects at three walking speeds.**

| Condition | Walking speed | Arch index (mean[std]) | Contact area (mm^2, mean[std]) |
| --- | --- | --- | --- |
| Control  (Control flat insole) | Slow | 0.22[0.04] | 9387[1612] |
|  | Normal | 0.21[0.06] | 9347[1756] |
|  | Fast | 0.21[0.05] | 9489[1724] |
| SCO  (Small calcaneus offloading insole) | Slow | 0.21[0.06] | 9004[1531] |
|  | Normal | 0.21[0.06] | 9259[1781] |
|  | Fast | 0.20[0.06] | 9212[1750] |
| LCO  (Larger calcaneus offloading insole) | Slow | 0.22[0.05] | 9607[1607] |
|  | Normal | 0.21[0.05] | 9545[1511] |
|  | Fast | 0.21[0.05] | 9752[1745] |
| SMHO  (Small metatarsal head offloading insole) | Slow | 0.20[0.05] | 9219[1513] |
|  | Normal | 0.20[0.06] | 9407[1745] |
|  | Fast | 0.20[0.05] | 9429[1557] |
| LMHO  (Large metatarsal head offloading insole) | Slow | 0.21[0.04] | 9379[1451] |
|  | Normal | 0.19[0.03] | 9258[1129] |
|  | Fast | 0.19[0.05] | 9554[1401] |
| LCOBS  (Both sides large calcaneus offloading insole) | Slow | 0.22[0.05] | 9412[1273] |
|  | Normal | 0.21[0.04] | 9444[1312] |
|  | Fast | 0.20[0.04] | 9573[1312] |
| Percentage difference with the P-value | | | |
| Condition | Walking speed | Arch index (Control value, no unit;  *difference from control, % and [P-value]*) | Contact area  (Control value, mm^2;  *difference from control, % and [P-value]*) |
| Control  (Control flat insole) | Slow | 0.22 | 9387 |
|  | Normal | 0.21 | 9347 |
|  | Fast | 0.21 | 9489 |
| SCO  (Small calcaneus offloading insole) | Slow | -2%[0.900] | -4%[0.001] |
|  | Normal | -2%[0.868] | -1%[0.556] |
|  | Fast | -7%[0.034] | -3%[0.101] |
| LCO  (Larger calcaneus offloading insole) | Slow | 3%[0.276] | 1%[0.526] |
|  | Normal | 2%[1.000] | 1%[0.936] |
|  | Fast | 1%[0.813] | 1%[0.379] |
| SMHO  (Small metatarsal head offloading insole) | Slow | -7%[0.149] | -3%[0.174] |
|  | Normal | -4%[0.262] | -1%[0.529] |
|  | Fast | -9%[0.035] | -2%[0.215] |
| LMHO  (Large metatarsal head offloading insole) | Slow | -9%[0.011] | -5%[0.031] |
|  | Normal | -14%[0.035] | -6%[0.049] |
|  | Fast | -14%[0.080] | -3%[0.136] |
| LCOBS  (Both sides large calcaneus offloading insole) | Slow | 0%[0.279] | -1%[0.529] |
|  | Normal | 1%[1.000] | 0%[0.726] |
|  | Fast | -3%[0.303] | 0%[0.685] |

## P-values were calculated for the five offloading conditions and the control condition for the 10 subjects at three walking speeds. P-values smaller than 0.05 were underlined.

**Please see the sentences below for text changes (added text highlighted in yellow):**

Line 229: *Only LCO and LCOBS showed a significant decrease in APPP in the region of interest at calcaneus when walking at all three speeds (P<0.05).*

Line 231: *When walking at slow, normal, and fast speed, LCO insoles could decrease more calcaneus APPP by a mean of -62% (range -57% to -68%, P<0.05) at the RoI than small calcaneus region offloading (SCO) insoles by a mean of -18% (-14% to -23%, P= 0.040, 0.108, and 0.264 respectively), but LCO insoles caused larger APPP increase of a mean of 23% (21% to 26%, P<0.05) in metatarsal head region at three walking speed.*

Line 234: *Calcaneus offloading insoles increased the APPP in calcaneus region (outside of the RoI) by a mean of 10% (range -1% to 17%) except large calcaneus offloading (LCO) insoles during slow walking while only SCO insoles at normal and fast walking speed observed significant increases (P=0.002 and 0.001). Calcaneus offloading insoles increased the APPP in metatarsal head region by a mean of 15% (range 4% to 26%) while only LCO insoles at three walking speed observed significant increases (P<0.05).*

Line 238: *Both small metatarsal head offloading (SMHO) and large metatarsal head offloading (LMHO) insoles could decrease the APPP at the metatarsal head RoI by a mean of -11% (range -3% to -21%, P>0.05) and LMHO decreased more during slow and fast walking.*

Line 240: *Metatarsal head offloading insoles increased the APPP in calcaneus region by a mean of 15% (range 11% to 19%) while the increase was only significant at fast walking speed (P<0.05), and they increased the APPP in metatarsal head region by a mean of 18% (range 8% to 34%, all P>0.05 except LMHO at normal walking speed).*

Line 262: *LCO insoles could decrease more calcaneus PTI by a mean of -70% (range -66% to -75%, P=0.000, 0.000, and 0.389) at the calcaneus RoI than SCO insoles by a mean of -20% (range -13% to -30%, P=0.025, 0.321, and 0.185), but they caused larger PTI increase in metatarsal head region during slow and normal walking. SCO insoles increased the PTI in calcaneus region by a mean of 10% (range 4% to 14%) while only observed the significant increase at normal walking speed (P=0.039). Calcaneus offloading insoles observed the PTI increase in metatarsal head region except SCO during normal walking speed (P>0.05). Metatarsal head offloading insoles decreased the PTI at the metatarsal head RoI by a mean of -23% (range -7% to -32%, P>0.05) but increased the PTI in metatarsal head region by a mean of 7% (range 3% to 12%, P>0.05).*

Line 282: *All offloading insoles except SMHO increased the heel strike velocity by a mean of 19% (range 1% to 32%, all P <0.05 except SCO at slow walking speed, SMHO at all walking speed, and LMHO at fast walking speed), as shown in Table 1. Toe-off velocity was observed to increase significantly by a mean of 15% (range 11% to 19%, P<0.05) when applying offloading insoles except SMHO.*

Line 305: *There was a statistical trend that showed decreases in the arch index for the SCO condition during fast walking (-7%, P=0.034), the SMHO condition during fast walking (-9%, P=0.035), and the LMHO condition during slow and normal walking (-9%, P=0.011; -14%, P=0.035). A statistical trend showed decreases in the total contact area for the SCO condition during slow walking (-4%, P=0.001) and the LMHO condition during slow and normal walking (-5%, P=0.031; -6%, P=0.049).*

Line 315: *When walking at slow and normal speeds, LCO observed a larger toe-off velocity than SCO, and the larger toe-off velocity corresponded to a larger APPP in metatarsal head region, see Table 1.*
